# New Mechanisms and Populations to Target in Binge Eating Treatment

**DOI:** 10.1001/jamanetworkopen.2025.25076

**Published:** 2025-08-01

**Authors:** Andrea K. Graham, Adrian Ortega, Amy Egbert

**Affiliations:** Center for Behavioral Intervention Technologies, Northwestern University Feinberg School of Medicine, Chicago, Illinois (Graham, Ortega); Department of Medical Social Sciences, Northwestern University Feinberg School of Medicine, Chicago, Illinois (Graham); Department of Preventive Medicine, Northwestern University Feinberg School of Medicine, Chicago, Illinois (Ortega); Department of Psychological Sciences, University of Connecticut, Storrs (Egbert).

In this study, Boutelle and colleagues^1^ tested the impact of combining a new intervention, regulation of cues (ROC), with behavioral weight loss treatment (BWL), on changes in binge eating and weight compared with cognitive behavioral therapy (CBT) among a sample of veterans. Boutelle et al^1^ focus on veterans given heightened rates of overweight and obesity and binge eating (based on military experiences and norms) in this population. Although CBT is the standard treatment for binge eating, it does not target weight, and although BWL is the standard treatment for weight, it does not directly target binge eating. As such, Boutelle and colleagues^1^ hypothesized that the addition of ROC, which targets the appetitive traits of food responsiveness and satiety responsiveness, would be effective for both weight loss and binge eating, given the impact of these traits on how an individual interacts with the food environment. Boutelle et al^1^ showed that ROC+BWL led to greater reductions in binge eating and weight during treatment compared with CBT, but differences in weight were no longer significant at the 6-month follow-up. Additionally, veterans who started treatment with binge eating disorder had lower odds of binge eating and greater reductions in weight when receiving ROC+BWL compared with CBT. There were no differences in feasibility or acceptability between treatments.

Existing research suggests that for individuals with binge eating, CBT is the predominant treatment for reducing binge eating episodes, and that BWL can be effective for reducing binge eating and body mass index in this population, but weight regain is common.^2^ Although the weight-related findings of Boutelle and colleagues^1^ support existing research, they are unique in that binge eating remained consistently lower in veterans who received ROC+BWL compared with those who received CBT. Furthermore, this study is important in (1) evaluating behavioral interventions among a specialized subpopulation (meaning, a specific group within a population that experiences a given disorder, in this case, veterans with overweight or obesity and binge eating) and (2) targeting a unique mechanism implicated in overeating behaviors. Research conducted among subpopulations of individuals with binge eating and that targets new mechanisms is warranted to increase the precision and impact of interventions—behavioral and otherwise—for binge eating and related behaviors.

Specific to subpopulations, there is growing attention in the eating disorders field to understanding and addressing binge eating among groups underrepresented and historically marginalized in eating disorder research,^3,4^ as well as for recognizing and addressing people’s full demographic profiles.^5^ For example, there is increasing awareness that the clinical presentation of binge eating and other disordered eating behaviors, as well as the mechanisms underlying their development and maintenance, may look different in people of varying sociodemographic profiles, including different races and ethnicities (eg, individuals who identify as Black, Latino, or South Asian), income levels and food security status, and military status. Yet, interventions specifically designed to address the unique needs of these subpopulations do not yet exist or are in their infancy, making the study by Boutelle and colleagues^1^ especially innovative in the population they targeted. Continued research studying interventions for specialized subpopulations with eating- and weight-related issues is warranted to achieve our ultimate goal of improving health for all.

A second innovation of the study by Boutelle and colleagues^1^ is its focus on novel intervention targets. Given that standard psychological treatments for binge eating, like CBT and interpersonal psychotherapy, are effective for some, but not all, individuals, it is imperative to design and test behavioral interventions that target novel mechanisms of action that can lead to improved outcomes. Here, the Boutelle et al^1^ targeted food responsiveness and satiety responsiveness, the efficacy of which makes ROC+BWL an important intervention option to move toward broader implementation and warrants evaluation of what implementation strategies are effective in these efforts. Additionally, their study provides an exemplar for other researchers to design additional interventions focused on novel mechanisms implicated in binge eating, like positive affect, shame, internalized weight bias, acculturative stress, and discrimination and exposure to racism.

In addition to the aforementioned future directions, other next steps emerge from this work by Boutelle et al.^1^ One is more research on understanding for whom, what, and when treatments work best, as this can improve the precision of interventions. In fact, studies that consider the timing of when interventions are introduced and delivered during the course of individuals’ treatment-seeking journeys, as well as in combination with studying which populations and under what conditions treatments are most effective, may be most impactful for optimizing treatment engagement and effectiveness.^6^ For example, Boutelle and colleagues could evaluate their trial data to identify when between-treatment differences in binge eating or weight change were most prominent during the course of treatment. Such an analysis can inform why treatments are working and when and areas for further optimization. Additional research that evaluates, as well as manipulates, the temporal impact of interventions, such as through micro–randomized trials, can improve the precision of binge eating treatments and inform new treatment targets.

Another key future direction from this work by Boutelle et al^1^ is regarding how digital components could interface with ROC treatment to improve both its precision and scale. Both treatments evaluated in the study by Boutelle and colleagues^1^ were delivered via weekly face-to-face group sessions (in person or via video conferencing software) by an interventionist. However, mental health clinician shortages^7^ mean that relying only on face-to-face intervention delivery will never reach all people in need. Therefore, a future direction for this work is testing whether components of ROC could be optimized through the use of digital app-based intervention. Digital augmentation and/or delivery would have the benefit of reaching more people in need, with the added benefit of providing in-the-moment support. In-the-moment intervention could be especially beneficial for practicing exposure exercises and addressing food responsiveness and satiety responsiveness, which are momentary experiences and vary across different environments. Digital tools, like ecological momentary assessments and sensors (eg, smartwatches, location tracking) could inform when individuals are experiencing moments of need, as well as be used to deliver just-in-time interventions, offering more precise intervention in the moments between sessions when practicing behavior change strategies can be most impactful. Furthermore, digital interventions can facilitate practice across diverse contexts to support more generalized learning and help individuals respond to cues across varying locations that confer risk for overeating. Such innovation is imperative to combat the multitude of prompts for problematic eating behaviors in our food environment.

To that end, while there is benefit in establishing individual-level interventions to support people in combating eating-related contextual cues, additional effort should be devoted to changing these contexts, through community-based or multilevel interventions, to support access to environments that promote healthier eating and safe physical activity. Doing so would bring forth a more high-impact solution to reducing the public health burden of binge eating and related disorders among all in need, particularly for subpopulations, like veterans, who are at heightened risk.
